# Pathogenesis of Acute Coronary Syndromes in Patients After COVID-19: An Optical Coherence Tomography Study

**DOI:** 10.3390/jcm14248895

**Published:** 2025-12-16

**Authors:** Krzysztof L. Bryniarski, Stanislaw Bartus, Jacek Legutko, Leszek Bryniarski, Pawel Gasior, Wojciech Wojakowski, Lukasz Rzeszutko, Artur Dziewierz, Wojciech Zasada, Tomasz Rakowski, Dawid Makowicz, Roman Wojdyla, Pawel Kleczynski, Ik-Kyung Jang

**Affiliations:** 1 Clinical Department of Interventional Cardiology, Saint John Paul II Hospital, Prądnicka 80, 31-202 Kraków, Poland; 2 Department of Interventional Cardiology, Institute of Cardiology, Jagiellonian University Medical College, 31-530 Kraków, Poland; 3 Clinical Department of Cardiology and Cardiovascular Interventions, University Hospital in Krakow, 30-688 Kraków, Poland; 4 2nd Department of Cardiology, Institute of Cardiology, Jagiellonian University Medical College, 30-688 Kraków, Poland; 5 Division of Cardiology and Structural Heart Diseases, Medical University of Silesia, 40-055 Katowice, Poland; 6 Healt Institute, State University of Applied Sciences in Krosno, 38-400 Krosno, Poland; 7 Center of Invasive Cardiology, Angiology and Electrotherapy, Intercard, 38-400 Krosno, Poland; 8 Cardiology Division, Massachusetts General Hospital—Harvard Medical School, Boston, MA 02114, USA; 9 Division of Cardiology, Kyung Hee University Hospital, Seoul 02447, Republic of Korea

**Keywords:** acute coronary syndromes, percutaneous coronary intervention, coronavirus-2019, optical coherence tomography, coronary plaque morphology

## Abstract

**Background:** Whilst COVID-19 mainly affects the lungs, multiple other organs were also involved—patients with COVID-19 were reported to be at higher risk of acute coronary syndromes (ACS). Importantly, results show that the risk of ACS may extend well beyond the acute phase of COVID-19 infection. In our study, we sought to investigate optical coherence tomography (OCT)-derived vascular changes, including the prevalence of plaque erosion in patients who had recent COVID-19. **Methods:** Patients with ACS were divided into two groups: those after COVID-19 infection during the past 12 months (post-COVID group) and those without known prior COVID infection (non-COVID group). We enrolled 35 patients in the post-COVID group and 35 patients in the non-COVID group. **Results:** The mean time from COVID infection to the imaging in the post-COVID group was 10 ± 1 months. There were no major differences in baseline demographic, clinical, or laboratory characteristics between the two groups. Erosion was the underlying pathology in one-third (34.3%) of the non-COVID group and in one-half (48.6%) of the post-COVID group, although the difference did not reach statistical significance. No calcified nodules were observed. The lipid core tended to be longer in the post-COVID group (9.1 ± 3.6 vs. 12.0 ± 1.9 mm; *p* = 0.005), and the prevalence of macrophages was higher in patients who had prior COVID-19 infection (48.6 vs. 74.3%; *p* = 0.027). **Conclusions:** Our OCT study demonstrated that patients with a prior COVID-19 infection tended to have a higher prevalence of plaque erosion and more vulnerable plaque morphology at the culprit lesion compared to those without a history of prior COVID-19 infection.

## 1. Introduction

The coronavirus pandemic in 2019 (COVID-19) was an unprecedented global event that resulted in over 6 million deaths [1]. COVID-19 negatively affected different areas of life, including healthcare systems around the world which were required to restructure and adapt to an entirely novel disease entity. Whilst COVID-19 mainly affected the lungs, multiple other organs were also involved. Patients with COVID-19 were reported to be at higher risk of cardiovascular disease, including myocardial injury and acute coronary syndromes (ACS) [2,3,4]. Although the exact mechanisms of ACS in COVID-19 patients are yet to be determined, they are thought to be related to destabilization of atherosclerotic plaques through systemic inflammatory responses, cytokine storm, and changes in immune cell polarization [3,5]. An alternative mechanism for ACS is endothelial damage and denudation in the presence of a hypercoagulable state. A recent optical coherence tomography (OCT) study demonstrated that patients several weeks after viral infection had more plaque ruptures, erosions, and diffuse lesions compared to those who did not suffer from COVID-19 [6].

A growing body of large-scale, high-quality studies has examined the sequelae associated with COVID-19 infection. The term post-COVID syndrome, also referred to as long COVID, was first introduced in 2020 [7]. It denotes a multisystem disorder that may develop even following relatively mild cases of acute COVID-19. Individuals affected by post-COVID syndrome frequently report a constellation of symptoms, most commonly including persistent fatigue, dyspnea, chest pain, and neuropsychiatric disturbances such as anxiety, depression, and cognitive impairment [7]. An increased incidence of cardiovascular manifestations and thromboembolic complications has also been observed in the post-acute phase of COVID-19 [8]. These findings, while concerning, were not entirely unexpected, as the detrimental effects of viral and bacterial infections on the cardiovascular system have been recognized in long-term follow-up studies well before the COVID-19 pandemic [5,9,10]. The recognition of post-COVID syndrome has therefore underscored the need for a deeper understanding of the long-term pathophysiological interactions between infectious diseases, systemic inflammation, and cardiovascular health. Xie et al. reported that patients up to 1 year after COVID-19 infection had an increased risk of cerebrovascular disorders, dysrhythmias, myocarditis, heart failure, and ischemic coronary artery disease, including acute myocardial infarction [11]. Similarly, in a systemic review and meta-analysis, Zuin et al. reported that during mean follow-up of almost 9 months ACS occurred in 3.5 cases per 1000 individuals with COVID-19 infection as compared to 2.02 cases per 1000 individuals in patients without prior COVID-19 infection [12]. These results show that the risk of ACS may extend well beyond the acute phase of COVID-19 infection. Some studies suggested that impaired endothelial function may be present up to 1 year after COVID-19 infection [13]. One of the mechanisms explaining those results may be change in coronary plaque characteristics and shift in the prevalence of factors triggering acute coronary syndromes. To our knowledge, no study assessed in vivo coronary plaque characteristics in patients presenting with ACS up to one year after COVID-19 infection. Thus, in our study, we sought to investigate vascular changes, including the prevalence of plaque erosion in patients who had recent COVID-19.

## 2. Materials and Methods

A total of 70 patients presenting with ACS were prospectively enrolled between May 2022 and May 2024 in St. John Paul II Hospital and University Hospital in Krakow. All patients underwent standard diagnosis and treatment as defined by the European Society of Cardiology Guidelines [14]. Patients were divided into two groups: those after COVID-19 infection during the past 12 months (post-COVID group) and those without known prior COVID infection (non-COVID group). Past COVID infections were verified in the patients’ medical records and through an interview. Only patients with a documented positive COVID-19 test during the previous year were included in the post-COVID group. Patients with any symptoms of flu which were not verified with a COVID-19 test during past two years and without at least one negative COVID-19 test taken during past year were excluded from the non-COVID group. Moreover, any known prior COVID-19 infection excluded patients from the non-COVID group. Patients with active infection (based on medical interview, white cell blood count, CRP levels, and additional tests including antigen test) were excluded from the study. One year phone follow-up was performed for all enrolled patients. Use of coronary stents or ballons as well as treatment after percutaneous coronary intervention was according to the operators and physicians discretion.

OCT imaging of the lesions was performed using the Ilumien OCT system (Abbott Vascular, Santa Clara, CA, USA). Pre-stent OCT images of the culprit lesions were analyzed at every 1 mm interval. The thrombectomy before OCT pullback was performed according to the operator’s discretion. Lipid-rich plaque was defined as a plaque with a maximum lipid arc > 90 degrees. Lipid index was calculated as the mean lipid arc multiplied by lipid length [15]. In addition, the presence of thin-cap fibroatheroma (TCFA), macrophage accumulation, cholesterol crystal, and microvessels was noted. TCFA was defined as a lipid-rich plaque with a fibrous cap thickness of ≤65 µm [15,16]. Macrophage accumulation on the OCT images was identified by granular structure with a high signal intensity within the plaque, accompanied by heterogeneous back shadows [17]. Cholesterol crystals were characterized as high-intensity, thin, linear structures. Microvessel was defined as small vesicular or tubular structures with a diameter of 50 to 300 µm. An area of low backscatter with sharp borders inside a plaque was defined as calcification [18,19]. The calcium index was calculated for each calcium deposit as the product of its calcium length and the mean calcium arc. Total calcium index was calculated as the sum of the calcium indices of each calcium deposit within the analyzed lesions [19]. OCT images were analyzed by 2 investigators who were blinded to the subject’s information. When there was a discordance between the readers, the consensus reading was obtained from a third independent investigator. The study was approved by the Institutional Review Board. Written informed consent was obtained from all the patients before enrolment. Participation in the study did not change the approach to the patients’ treatment in comparison to standard management.

Categorical data are presented as counts and percentages (%). The normality of distributions of continuous variables was examined using a Kolmogorov–Smirnov test. The mean and standard deviation are reported for normally distributed data, while the median (with 25th and 75th percentiles) is reported for data that are not normally distributed. Fisher’s exact test or Chi-square test was used to analyze categorical variables, and a Student’s *t* test or Mann–Whitney U test for continuous variables. Statistical significance was defined as *p* < 0.05. All statistical analyses were performed with SPSS 29.0 (SPSS Inc., Chicago, IL, USA).

## 3. Results

Overall, we enrolled 35 patients in the post-COVID group and 35 patients in the non-COVID group. There were no differences in baseline demographic, clinical, or laboratory characteristics between the two groups, except that post-COVID patients tended to be older and had higher eGFR and lower troponin T on admission (Table 1). Most patients were male and were slightly overweight. The mean time from COVID infection to the imaging in the post-COVID group was 10 ± 1 months. Procedural data were also comparable between the two groups (Table 2).

In both groups left anterior descending artery was the most frequent culprit vessel. Percutaneous coronary intervention with placement of one stent was performed in most cases. Mean lesion length in the non-COVID group was 17.9 ± 7.1 as opposed to mean lesion length of 19.4 ± 4.9 mm in the post-COVID group (*p* = 0.185). Thrombectomy was performed in approximately one-fourth of all cases and about one-third of patients had multivessel disease. Glycoprotein IIb/IIIa inhibitors were given to one (1.4%) patient in both groups (*p* = NS). In all procedures unfractionated heparin adjusted to weight was used.

**Figure 1:**
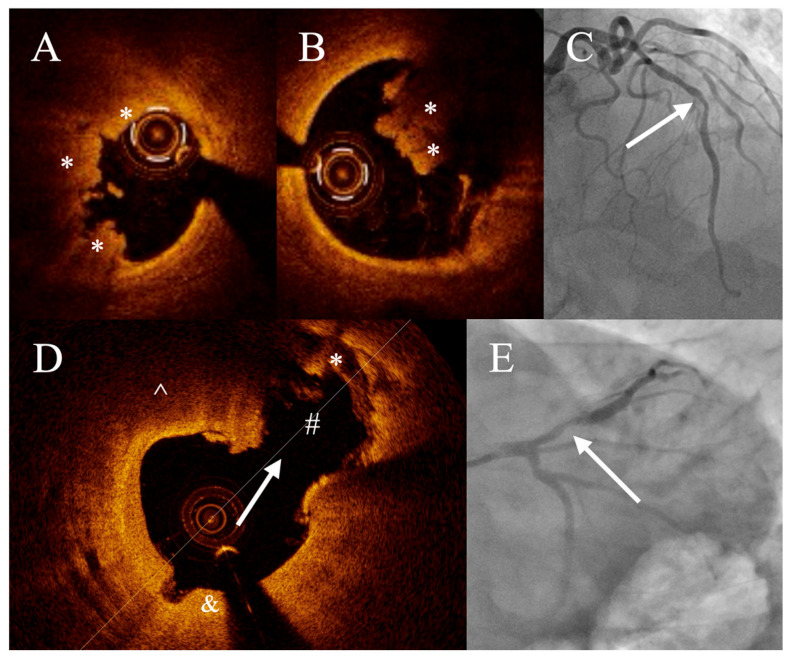
Optical coherence findings in patients with acute coronary syndrome. Patient with acute coronary syndrome (ACS). The OCT (**A**,**B**) was performed in lesion (arrow) in left anterior descending artery (**C**). OCT revealed thrombus (*), however no plaque rupture was observed—image suggestive of plaque erosion (erosion is described as presence of attached thrombus overlying an intact and visualized plaque). Second OCT (**D**) shows plaque rupture (arrow) in lesion in left anterior descending artery (**E**). Cavity of ruptured plaque is marked with #. Moreover, both lipid plaque (^) and fibrous plaque (&), as well as thrombus (*) are visible in the image.

**Figure 2:**
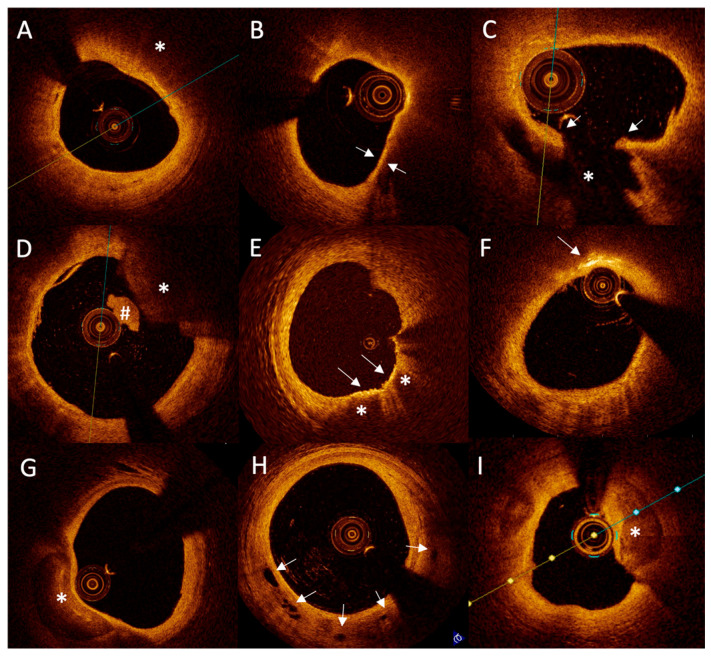
Representative images of chosen optical coherence tomography findings. Lipid plaque is characterized as signa poor regions (*) with overlying signal rich bands (**A**). Thin cap fibroatheroma is defined as a lipid plaque occupying more than >90° in circumference and with fibrous cap thickness (arrows) less than a set threshold (usually 65 μm or 80 μm) (**B**). Plaque rupture is defined as disruption of fibrous cap (arrows) with visible cavity within the plaque ((**C**); *). Red thrombus is described as highly backscattering structure with high attenuation ((**D**); *), whereas white thrombus is less backscattering and has lower attenuation ((**D**), #). Macrophage accumulation on the OCT image is described as granular structure with a high signal intensity within the plaque ((**E**), arrow), accompanied by heterogeneous back shadows ((**E**), *). Cholesterol crystals are characterized as high-intensity, thin, linear structures (arrow, (**F**)). An area of low backscatter with sharp borders inside a plaque was defined as calcification ((**G**), *). Microvessel was defined as small vesicular or tubular structures with a diameter of 50 to 300 µm ((**H**), arrows). Calcification protruding to the lumen is described as calcific nodule ((**I**); asterisk). Partially adapted from Legutko et al. [20].

Erosion was the underlying pathology in one-third (34.3%) of the non-COVID group and in one-half (48.6%) of the post-COVID group, although the difference did not reach statistical significance (Table 3, Figure 1 and Figure 2). No calcified nodules were observed. The lipid core tended to be longer in the post-COVID group, and the prevalence of macrophages was higher in patients who had prior COVID-19 infection. We observed a trend toward thinner fibrous cap in COVID-19 survivors, but the results did not reach statistical significance.

All of the patients after percutaneous coronary intervention were on dual antiplatelet therapy. In the non-COVID group, 9 patients (25.7%) were given clopidogrel, prasugrel was given to 13 patients (37.1%), and ticagrelor was given to 13 (37.1%) patients. In the post-COVID group 13 patients (37.1%) were given clopidogrel, prasugrel was given to 16 patients (45.7%), and ticagrelor was given to 6 (17.1%) patients. Those differences were not statistically significant (*p* = 0.303; *p* = 0.467; *p* = 0.060, respectively). Oral anticoagulants were given to one patient (1.4%) in the post-COVID group, and new oral anticoagulants were given to six patients (17.1%) in the non-COVID group and four patients (11.4%) in the post-COVID group (*p* = 0.495). In the non-COVID group, oral anticoagulants were given to patients due to AF/AFL (six patients had AF/AFL in their medical history). In case of the post-COVID group, two patients had AF/AFL in their medical history, another two had new onset of AF/AFL, and one had history of recent thromboembolism.

Clinical follow-up data were available for all the patients. In each group, one patient had to undergo repeat revascularization; no deaths were observed.

## 4. Discussion

To the best of our knowledge, this is the first study to focus on OCT-derived coronary plaque characteristics in post-COVID patients presented with ACS. Importantly, in our study we have included patients a long time after the acute COVID-19 phase, with average time from COVID infection reaching 11 months. In this study, we have observed (1) a trend towards a higher prevalence of plaque erosion in patients with previous COVID-19 infection, and (2) a higher prevalence of macrophages within the atheroma, as well as longer lipid lesions in patients with prior COVID-19 infection.

Although COVID-19 was identified as an independent risk factor for ACS during the first several weeks after infection, studies reported that the increased risk of cardiovascular events extends well beyond the first months after infection [8,11,12,21]. Xie yan et al., in a study involving 153,760 patients after COVID-19, 5,637,647 patients in a contemporary control, and 5,859,836 patients in a historical cohort, demonstrated that people after COVID-19 exhibited greater risk of acute coronary disease and myocardial infarction [11]. In Zhang et al. meta-analysis which included nearly 10 million individuals Authors showed that patients after COVID-19 as opposed to patients without COVID-19 infection had higher risk of, among others, thromboembolic disorders (HR 3.12 (1.60, 6.08)), coronary heart disease (HR 1.61 (1.13, 2.31)), myocarditis (HR 6.11 (4.17, 8.94)), and heart failure (HR 1.72 (1.15, 2.59) [21].

Importantly, consequences after COVID-19 may have a broad complications both in life expectancy of COVID-19 survivors as well as in their economic productivity.

The biological and physiological mechanisms that explain the relationship between COVID-19 infection and the subsequent development of cardiovascular diseases during the post-acute or recovery phase remain incompletely understood. While it is evident that individuals recovering from COVID-19 may experience a range of cardiovascular complications—such as myocarditis, arrhythmias, or ACS—the precise pathways through which the virus or the body’s immune response contributes to these outcomes have not yet been fully elucidated. Infections, including those of the respiratory tract, can trigger pro-inflammatory reactions, such as the release of interleukins (ILs) that activate inflammatory cells and destabilize atherosclerotic plaques. At the same time, neutrophils and monocytes can also be activated, leading to endothelial damage and a prothrombotic state. This was confirmed in some COVID-19 studies which suggested that this virus has a direct effect on endothelial cells and that the exaggerated inflammatory response as cytokine storm is likely to precipitate cardiovascular events through ACE2 receptor downregulation, platelet activation, and hypercoagulability [22]. These changes may result in a perfect storm for plaque rupture and plaque erosion. Indeed, autopsy studies reported inflamed plaques in COVID-19-infected coronary arteries [23]. In Eberhardt et al.’s study, respiratory syndrome coronavirus 2 (SARS-CoV-2) viral RNA was detectable and replicated in coronary lesions taken at autopsy from severe COVID-19 cases [23]. They showed that the virus replicated in macrophages within human coronary arteries of patients who died from severe COVID-19. Moreover, SARS-CoV-2 exhibited a stronger tropism for arterial lesions than adjacent perivascular fat. The virus induced an inflammatory response in cultured macrophages and human atherosclerotic vascular explants with secretion of cytokines known to trigger cardiovascular events.

Long-term effects of COVID-19 infections, including viral antigen persistence, immune mechanisms, and dysregulations of autoimmunity may lead to persistent endothelial and vascular dysfunction as well as residual inflammation [24]. The interplay between all these biological and mechanical conditions can induce atheromatous plaque erosion or rupture, resulting in coronary thrombosis and ACS [25]. In our study we did not find differences in rate of plaque erosion and rupture between patients with and without previous COVID-19 infection. However, we observed a trend towards a greater prevalence of plaque erosion in patients after COVID-19 infection. Inflammation, endothelial damage associated with plaque erosion, and prothrombotic activation of the coagulation cascade may change the balance between those two pathological mechanisms, causing ACS. In the case of COVID-19, the denudation of the coronary endothelium with subsequent exposure of the subendothelial matrix, promoting platelet adhesion and thrombus formation in the environment of a hypercoagulable state, could be a plausible explanation for the increased rate of ACS caused by plaque erosion in COVID-19 patients. However, bigger studies are needed to explore this hypothesis. To our knowledge, no studies have assessed the incidence of OCT-derived plaque rupture and erosion in patients with viral infection. If confirmed in larger studies, this could change the approach to patients with ACS and after COVID-19. As demonstrated by several recent studies, elucidating the exact pathophysiological mechanisms underlying ACS enables clinicians to take a more tailored approach in the ACS setting [26]. Plaque erosion was described as a distinct pathogenic and clinical entity as compared to plaque rupture [27]. It has an intact vessel cap and larger vessel lumen when compared to plaque rupture [28]. What is more, platelet-rich thrombus is more frequent in this entity. Importantly, when compared to patients with plaque rupture, those with plaque erosion have a better risk profile and outcomes [28]. Hence, different management of patients with plaque erosion and rupture may be proposed. For instance, the EROSION study provided important insights into the treatment of patients presenting with ACS secondary to plaque erosion. During 4 years follow-up of this study, out of 52 patients only 11 (21%) patients had target lesion revascularization [29]. Moreover, no deaths, heart failures, strokes, recurrent myocardial infarctions, unstable angina induced hospitalizations, or coronary artery bypass graftings were observed. Hence, the study demonstrated that, in patients with plaque erosion, intensive antithrombotic therapy alone may achieve favorable outcomes without the need for stent implantation [30]. If higher prevalence of plaque erosion is indeed confirmed in larger studies, this could insinuate the more frequent use of OCT in post-COVID patients in order to determine the best approach.

In our analysis, we observed a greater prevalence of macrophages and longer lipid lesions in patients after COVID-19 infection. Patients with ACS often showed a thinner fibrous cap, greater prevalence of macrophages, and a larger lipid core when compared to patients with stable angina—those are the hallmarks of the coronary plaque vulnerability [20]. Similarly to our results, in a computed tomography angiography (CCTA) study, patients 4.5 months after COVID-19 infection had higher levels of pericoronary inflammation as opposed to those without prior COVID-19 infection [31]. Of importance, pericoronary inflammation has been shown to predict the risk of acute coronary events. Another CCTA study which included 803 subjects found that patients after COVID-19 infection had an increased risk of high-risk plaques and a more rapid progression of percent atheroma volume of coronary plaques [32]. In this study the authors found that the influence of COVID-19 infection on coronary plaque advancement was partially mediated by coronary inflammation. In theory, COVID-19 could play a role in aggravation of preexisting coronary artery disease.

Some data suggested that patients after COVID-19 and PCI have higher risk of stent restenosis [33]. Persistent inflammatory processes may be associated with vascular remodeling and the fibrosis process. Although all of the patients in our study had one year follow-up, the study did not have sufficient statistical power to assess major adverse cardiac events.

Although non significant, we observed a trend toward more previous PCIs in the post-COVID group. So far, no studies including a large cohort of patients address differences in plaque characteristics between patients with first ACS and with recurrent ACS [34]. However, it is well known that patients with recurrent ACS have worse long-term prognosis [35,36]. Our study was not powered to assess MACE in both groups; however, it is possible that those with recurrent ACS may have worse outcomes regardless of their COVID infection status. Noteworthily, the question remains if COVID-19 may contribute to a higher number of myocardial infarctions in individuals who have already experienced ACS. This should be addressed in larger studies.

Several limitations should be noted. First, this was an explorative study with a small number of patients. This study was designed to test the hypothesis that the prevalence of erosion in COVID patients would be higher due to endothelial damage and hypercoagulable status. Although the difference did not reach statistical significance, there was a trend toward greater prevalence of plaque erosion. Second, upon admission, only morphology and CRP/hsCRP levels were tested; we did not measure levels of IL-6 or any other cytokines. Moreover, patients had either CRP or hsCRP levels checked at the admission, and thus we did not include this parameter in analysis.

## 5. Conclusions

Our OCT study demonstrated for the first time that a clinically apparent COVID-19 infection may be associated with changes in the composition and vulnerability of coronary atherosclerotic lesions that may persist long after the infection has subsided. We have observed a trend towards greater prevalence of plaque erosion in patients with previous COVID-19 infections, as well as a higher prevalence of macrophages and longer lipid plaques in patients post a clinically relevant COVID-19 infection. This is a preliminary study, and as such, its results must be treated with caution. The intriguing notion that a COVID-19 infection may affect the composition of atherosclerotic plaques deserves further investigation on larger cohorts. If confirmed, our observations could change our understanding of the mechanisms behind ACS in COVID-19 survivors.

## Figures and Tables

**Table 1:** Baseline characteristics.

	Non COVID n = 35	Post COVID n = 35	*p* Value
Age, years	55.7 ± 12.0	65 ± 9.2	<0.001
Sex (Male), n (%)	25 (71.4)	20 (57.1)	0.212
BMI, kg/m^2^	29.4 ± 5.3	27.3 ± 4.5	0.097
Diabetes mellitus, n (%)	7 (20.0)	6 (17.1)	0.759
Hypertension, n (%)	24 (68.6)	21 (60.0)	0.454
Hyperlipidemia, n (%)	27 (77.1)	29 (82.9)	0.550
Previous PCI, n (%)	6 (17.1)	13 (37.1)	0.060
Previous CABG, n (%)	0 (0.0)	0 (0.0)	-
Previous MI, n (%)	5 (14.3)	8 (22.9)	0.356
Stroke, n (%)	2 (5.7)	2 (5.7)	-
AF/AFL, n (%)	6 (17.1)	2 (5.7)	0.133
CKD, n (%)	7 (20.0)	5 (14.3)	0.555
EF, %	51.5 ± 10.6	46.8 ± 10.6	0.065
Current smoker, n (%)	11 (31.4)	14 (40.0)	0.454
Laboratory data			
Total cholesterol, mmol/L; mg/dL	4.7 ± 1.2; 182 ± 46	4.8 ± 1.4; 186 ± 54	0.753
LDL-C, mmol/L; mg/dL	2.9 ± 0.9; 112 ± 35	2.9 ± 1.2; 112 ± 46	0.845
HDL-C, mmol/L; mg/dL	1.2 ± 0.5; 46 ± 19	1.4 ± 0.4; 54 ± 15	0.144
Triglyceride, mmol/L; mg/dL	1.4 ± 0.6; 124 ± 53	1.3 ± 0.5; 115 ± 44	0.667
Hgb, g/dL	13.3 ± 1.7	13.4 ± 2.1	0.898
WBC, ×1000/μL	10.8 ± 2.9	9.8 ± 2.8	0.156
eGFR, mL/min,1.73 m^2^	71.4 ± 14.9	81.5 ± 20.0	0.026
Troponin hs (admission), ng/L	2360 (1200; 4068)	2055 (1000; 3500)	0.004
Presentation—STEMI, n (%)	13 (37.1)	16 (45.7)	0.530

Values are mean ± standard deviation (SD) or n (%). AF/AFL indicates atrial fibrillation/atrial flutter; BMI, body mass index; CABG, coronary artery bypass graft; CKD, chronic kidney disease; EF, ejection fraction; HDL-C, high-density lipoprotein cholesterol; Hgb, hemoglobine; LDL-C, low-density lipoprotein cholesterol; MI, myocardial infarction; PCI, percutaneous coronary intervention; STEMI, ST segment elevation myocardial infarction; WBC, white blood cells.

**Table 2:** Procedural data.

	Non COVID n = 35	Post COVID n = 35	*p* Value
Culprit vessel			0.483
LAD, n (%)	21 (60.0)	16 (45.7)	
RCA, n (%)	10 (28.5)	14 (40.0)	
Cx, n (%)	4 (11.5)	5 (14.3)	
POBA, n (%)	1 (2.9)	4 (11.4)	0.088
Number of stents			0.226
1, n (%)	25 (71.4)	22 (62.9)	
2+, n (%)	9 (25.7)	9 (25.7)	
Thrombectomy, n (%)	11 (31.4)	7 (20.0)	0.423
Multivessel disease, n (%)	15 (42.9)	13 (37.1)	0.626

Values are mean ± standard deviation (SD) or n (%). Cx indicates circumflex coronary artery; LAD, left anterior descending coronary artery; POBA, plain old ballon angioplasty; RCA, right coronary artery.

**Table 3:** OCT findings.

	Non COVID n = 35	Post COVID n = 35	*p*
Underlying pathology (ACS)			0.225
Rupture, n (%)	23 (65.7)	18 (51.4)	
Erosion, n (%)	12 (34.3)	17 (48.6)	
Lesion length, mm	17.9 ± 7.1	19.4 ± 4.9	0.185
Lipid rich, n (%)	21 (60.0)	22 (62.9)	0.806
Lipid core length, mm	9.1 ± 3.6	12.0 ± 1.9	0.005
Mean lipid arc, °	170 ± 39	181 ± 34	0.451
Max lipid arc, °	258 ± 70	274 ± 55	0.601
Lipid index	1540 ± 616	2200 ± 560	0.060
TCFA, n (%)	10 (28.6)	11 (31.4)	0.571
FCT, mm	0.85 ± 0.27	0.71 ± 0.25	0.089
Calcification, n (%)	16 (45.7)	22 (62.9)	0.150
Macrophages, n (%)	17 (48.6)	26 (74.3)	0.027
Microchannels, n (%)	12 (34.3)	11 (31.4)	0.799
Cholesterol crystals, n (%)	11 (31.4)	15 (42.9)	0.322

Values are mean ± standard deviation (SD) or n (%). ACS indicates acute coronary syndrome; FCT, fibrous cap thickness; TCFA, thin-cap.

## Data Availability

The data presented in this study are available on request from the corresponding author due to constraints in approval from Ethic Committee and sponsor of the study.
